# Exploring the relationship between vitamin D deficiency and comorbid heart disease in Americans with mood disorders: a cross-sectional nationwide study

**DOI:** 10.3389/fpsyt.2023.1256126

**Published:** 2023-10-23

**Authors:** Maleeha Habib, Sanobar Jaka, Sandesh Pokhrel, Albulena Sejdiu, Archna Patel, Sreshatha Vashist, Abimbola Arisoyin, Meenal Pathak, Anil K. Bachu, Senthil Vel Rajan Rajaram Manoharan, Raja Mogallapu, Rikinkumar S. Patel

**Affiliations:** ^1^St. George’s University School of Medicine, True Blue, St. George’s, Grenada; ^2^Department of Population Health, School of Medicine, New York University, New York, NY, United States; ^3^Nepal Medical College Teaching Hospital, Kathmandu, Nepal; ^4^Department of Psychiatry, Ss. Cyril and Methodius University in Skopje, Skopje, North Macedonia; ^5^Department of Psychiatry, NIMS Medical College and Hospital, NIMS University, Jaipur, India; ^6^N.C. Medical College and Hospital, Panipat, India; ^7^Psychiatry Department, College of Medicine, University of Lagos, Lagos, Nigeria; ^8^Department of Psychiatry and Behavioral Health, College of Medicine, The Pennsylvania State University, Hershey, PA, United States; ^9^Department of Psychiatry, College of Medicine, University of Arkansas for Medical Sciences, Little Rock, AR, United States; ^10^Department of Psychiatry and Behavioral Neurobiology, Heersink School of Medicine, University of Alabama at Birmingham, Birmingham, AL, United States; ^11^Department of Behavioral Medicine and Psychiatry, School of Medicine, West Virginia University, Morgantown, WV, United States; ^12^Department of Psychiatry and Behavioral Sciences, School of Medicine, Duke University, Durham, NC, United States

**Keywords:** mood disorder, heart disease, vitamin D deficiency, major depressive disorder, bipolar disorder, tobacco-related disorders, trauma-related disorders

## Abstract

**Objective:**

This study aimed to explore the relationship between vitamin D deficiency and comorbid heart disease in adult inpatients with mood disorders (depressive and bipolar disorders).

**Methods:**

A cross-sectional investigation was carried out employing the nationwide inpatient dataset, which encompassed 910,561 adult inpatients aged 18 to 50 years diagnosed with depressive and bipolar disorders. Additionally, the sample was categorized based on the presence of comorbid heart disease. We utilized a logistic regression model to assess the odds ratio (OR), pertaining to demographic features and coexisting medical conditions in relation to comorbid heart disease.

**Results:**

Comorbid heart disease was present in 1.3% of inpatients with mood disorders; they were middle-aged (mean age 42.7 years) men and White individuals. Inpatients with depressive disorder had a higher risk of comorbid heart disease (OR 1.19, 95% CI 1.15–1.24) compared to those with bipolar disorders. Inpatients with comorbid heart disease had a higher prevalence of medical and psychiatric comorbidities. The prevalence of vitamin D deficiency was 2.3% in mood disorders but higher in those with comorbid heart disease (2.9%). Vitamin D deficiency showed a notable correlation with comorbid heart disease, resulting in a 26% increased risk in the unadjusted regression model (OR 1.26, 95% CI 1.13–1.40). However, after accounting for potential confounding factors, including comorbidities, the risk did not exhibit statistical significance (OR 1.08, 95% CI 0.97–1.21). Among psychiatric comorbidities, trauma-related (OR 1.22, 95% CI 1.17–1.28) and tobacco-related (OR 1.31, 95% CI 1.26–1.37) disorders had a higher risk of association with comorbid heart disease.

**Conclusion:**

Middle-aged men with depressive disorders and from low-income families had a higher risk of developing comorbid heart disease. Trauma-related and tobacco-related disorders were associated with an increased risk by 20–30% for comorbid heart disease in inpatients with mood disorders. Vitamin D deficiency was not associated with the risk of comorbid heart disease after controlling demographics and comorbid cardiovascular risk factors.

## 1. Introduction

In the United States (US), mood disorders constitute a noteworthy issue, with approximately 21.4% of adults indicating they experienced at least one mood disorder over the course of their lives (1, 2). The most recent findings from the 2021 National Survey on Drug Use and Health indicate that 8.3% of adults aged 18 years and older, spanning all racial backgrounds, encountered a depressive episode in the previous year (1). The annual prevalence of bipolar disorder among adults in the US stands at 2.8% (3). Mood disorders, including depressive episodes and bipolar disorder, have far-reaching consequences on individuals’ lives. They often contribute to distorted self-image, reduced productivity, and strained interpersonal relationships, causing significant distress in daily functioning. Moreover, these disorders are associated with an increased risk of substance use and suicidal ideation, with a mortality rate of 14.5 per 100,000 deaths (4).

The relationship between chronic medical conditions and coexisting psychiatric comorbidities, such as mood disorders, is complex and varies from person to person (5). A recent research study revealed a range of patterns, including situations in which patients experience depression or anxiety following a diagnosis of a chronic disease, instances where individuals with preexisting mental health conditions are at a greater risk of developing chronic diseases, and a cyclic interplay where each condition can mutually influence and worsen the other (5).

In 2017, a meta-analysis examined the coexistence of heart disease in individuals with severe mental illness (SMI), encompassing patients with schizophrenia, depressive disorder, and bipolar disorder (BPD) (6). The findings indicated that the prevalence of comorbid heart disease was 11.7% in individuals with depressive disorder and 8.4% in those with bipolar disorder (6). A separate meta-analysis, centered on examining depression as a risk factor for the co-occurrence of heart disease, identified a noteworthy association between these two conditions, revealing an odds ratio of 2.52 (7). Cardiovascular diseases (CVDs) constitute a significant factor in approximately 35–40% of mortality cases among individuals diagnosed with bipolar disorder (BPD). Moreover, individuals with BPD who experience CVD-related fatalities tend to do so at a relatively younger age when compared to those without bipolar disorder (8). The exact mechanism underlying the development of heart disease in individuals with mood disorders remains unclear. However, potential factors include dysfunctions in the autonomic nervous system, hypothalamic–pituitary–adrenal axis dysregulation, increased platelet reactivity, elevated inflammation, medication effects, poor dietary choices, lack of physical exercise, alcohol or substance abuse, smoking, sleep disturbances, and genetic factors (7). Another theory posits that heart disease and mood disorders share several comorbidities, such as hypertension, diabetes mellitus, hyperlipidemia, systemic inflammation, and vitamin D deficiency as both the heart and the brain require high energy and lack regenerative abilities after tissue injury (9, 10).

Despite the limitations of earlier studies, there is a prevailing belief that vitamin D deficiency may be associated with psychiatric disorders primarily because vitamin D receptors are present in brain regions associated with depression (9, 11). It is also recognized that cell-mediated immunity and inflammation are associated with depression, comorbid heart disease, and vitamin D deficiency (12). Furthermore, a notable connection has been established between elevated systemic inflammatory markers in individuals suffering from both depression and concurrent heart disease. Considering that vitamin D is endowed with anti-inflammatory and antioxidant attributes, it could potentially serve as a crucial element in the management of depression and coexisting heart disease (12, 13).

These findings collectively highlight the interplay between mood disorders, heart disease, and vitamin D deficiency. Further research is warranted to elucidate the underlying mechanisms and explore potential therapeutic implications. There is limited literature and inconclusive findings on this topic demonstrated by multiple systematic reviews (14–16). Hence, our study first aimed to delineate the differences in demographics and comorbidities in inpatients with mood disorders by comorbid heart disease. Second, it aimed to evaluate the association of demographics and comorbidities including vitamin D deficiency with comorbid heart disease in mood disorder inpatients.

## 2. Materials and methods

### 2.1. Study sample

We conducted a cross-sectional study using the Nationwide Inpatient Sample (NIS 2018–2019) that consists of patient records from non-federal community hospitals across 48 states and the District of Columbia in the US (17). NIS is a publicly available de-identified dataset from the Agency for Healthcare Research and Quality (AHRQ), and institutional review board permission was not required as per the US Department of Health and Human Services (17).

Our study sample included 910,561 adult inpatients (age 18–50 years) with a primary discharge diagnosis of depressive and bipolar disorders. The study sample was further grouped based on co-diagnosis of coronary atherosclerosis and heart disease, and we used the term “comorbid heart disease” in this study.

### 2.2. Variables

The demographic variables included in the study were age, sex (male or female), race (White, Black, Hispanic, and others), and median household income (per national percentile). Comorbidities are co-diagnosed in the patient records. We included both psychiatric and medical comorbidities: anxiety disorders, trauma-related disorders, neurodevelopmental disorders, tobacco-related, alcohol-related and substance-related disorders, hypertension, diabetes, and obesity. Vitamin D deficiency was identified in the NIS using the International Classification of Diseases 10th revision (ICD-10) codes E55.9 or E56.0.

### 2.3. Statistical analysis

We conducted a comparative analysis of demographic characteristics and comorbidities among inpatients diagnosed with mood disorders (including depressive and bipolar disorders) based on the presence of comorbid heart disease. This analysis involved utilizing descriptive statistics and Pearson’s chi-square test. Subsequently, we employed a binary logistic regression model to assess the odds ratio (OR) for variables associated with comorbid heart disease, considering both demographic predictors and comorbid risk factors. Our approach involved employing three independent binary regression models: Model 1, which examined the unadjusted OR; Model 2, which adjusted OR for demographics; and Model 3, which further adjusted OR for demographics, diagnosis, and comorbidities. This method allowed us to explore potential interactions among various variables concerning comorbid heart disease in individuals with mood disorders. All statistical analyses were carried out using the Statistical Package for the Social Sciences (SPSS) by IBM Corp. located in Armonk, NY. The statistical significance threshold was set at a two-sided *p*-value of <0.05.

## 3. Results

A total of 910,561 inpatients with mood disorders were included in the study. A majority of the study inpatients were young adults, 18–35 years (60.7%), women (52.4%), and White in ethnicity (68.3%).

The prevalence of comorbid heart disease in inpatients with these mood disorders was approximately 1.3%. The mean age of mood disorder inpatients with comorbid heart disease was significantly higher (42.7 years) than those without (32.4 years). A significantly higher proportion of the mood disorder inpatients with comorbid heart disease were middle-aged with 36–50 years (85.2% vs. 38.7%), male (61.2% vs. 47.4%), and White ethnicity (71.4% vs. 68.3%). Furthermore, a statistically significant distinction was evident when comparing cohorts, with a higher prevalence of comorbid heart disease observed among inpatients with a median household income below the 50th percentile.

Mood disorder inpatients with comorbid heart disease had a significantly higher prevalence of medical comorbidities including hypertension (52.2%), diabetes (17.3%), obesity (18.8%), and vitamin D deficiency (2.9%) compared to those without. Additionally, they had a higher prevalence of psychiatric comorbidities such as anxiety disorders (43.4%), trauma-related disorders (26.0%), tobacco-related disorders (53.5%), and alcohol-related disorders (23.9%). The majority of primary discharge diagnoses among inpatients with comorbid heart disease was depressive disorder (63.8%).

In inpatients with mood disorders, the prevalence of vitamin D deficiency stood at 2.3%, with a notable contrast observed in individuals with comorbid heart disease, where the prevalence was higher at 2.9%, as indicated in Table 1.

**Table 1:** Differences in demographics and comorbidities in inpatients with mood disorder by comorbid heart disease.

Variable	Total	Comorbid heart disease		*p*-value
		No	Yes	
Number of inpatients	910,561	898,826	11,735	–
Mean age (SD)	32.6 (9.6)	32.4 (9.5)	42.7 (6.9)	<0.001
Age, in %				
18–35 years	60.7	61.3	14.8	<0.001
36–50 years	39.3	38.7	85.2	
Sex, in %				
Male	47.6	47.4	61.2	<0.001
Female	52.4	52.6	38.8	
Race, in %				
White	68.3	68.3	71.4	<0.001
Black	15.9	15.9	17.9	
Hispanic	9.8	9.9	7.5	
Other	5.9	6.0	3.2	
Median household income, in %				
Below 50th percentile	60.6	60.4	72.7	<0.001
Above 50th percentile	39.4	39.6	27.3	
Primary discharge diagnosis, in %				
Depressive disorders	61.1	61.1	63.8	<0.001
Bipolar disorders	38.9	38.9	36.2	
Psychiatric comorbidities, in %				
Anxiety disorders	56.6	56.6	43.4	<0.001
Trauma-related disorders	22.2	22.2	26.0	<0.001
Neurodevelopmental disorders	10.5	10.5	6.2	<0.001
Tobacco-related disorders	42.8	42.7	53.5	<0.001
Alcohol-related disorders	20.3	20.2	23.9	<0.001
Substance-related disorders	41.3	41.3	40.3	0.033
Medical comorbidities, in %				
Hypertension	16.2	15.7	52.2	<0.001
Diabetes	4.5	4.3	17.3	<0.001
Obesity	9.5	9.4	18.8	<0.001
Vitamin D deficiency	2.3	2.3	2.9	<0.001

Vitamin D deficiency displayed a noteworthy correlation with comorbid heart disease, with the risk rising by 26% in both the unadjusted Model 1 and the adjusted Model 2 (controlling for demographics). However, in the final adjusted Model 3, where additional factors were considered (adjusted odds ratio 1.08, 95% confidence interval 0.97–1.21), the association’s risk was no longer statistically significant. The most influential factors predicting comorbid heart disease were age, with patients aged 36–50 years showing a substantial association [adjusted odds ratio (aOR) 6.12, 95% CI 5.79–6.47], and gender, with males exhibiting a higher risk (OR 1.79, 95% CI 1.72–1.86) in comparison to their counterparts. Regarding other demographic predictors, race/ethnicity did not show a significant association with comorbid heart disease. However, individuals from low-income households, with incomes below the 50th percentile, had an elevated risk of comorbid heart disease (OR 1.47, 95% CI 1.40–1.53).

Among the comorbidities, hypertension had the strongest association with comorbid heart disease (OR 2.86, 95% CI 2.75–2.98), followed by diabetes (OR 1.93, 95% CI 1.82–2.03) and obesity (OR 1.54, 95% CI: 1.46–1.62). Among psychiatric comorbidities, trauma and stress-related disorders (OR 1.22, 95% CI 1.17–1.28), and tobacco-related disorders (OR 1.31, 95% CI 1.26–1.37) had a higher risk of association with comorbid heart disease as shown in Table 2.

**Table 2:** Predictors for comorbid heart disease in inpatients with mood disorders.

Variable	Odds ratio (95% confidence interval)		
	Model 1	Model 2	Model 3
Vitamin D deficiency	1.26 (1.13–1.40)	1.26 (1.13–1.40)	1.08 (0.97–1.21)
Age			
18–35 years	–	Reference	Reference
36–50 years	–	8.88 (8.42–9.37)	6.12 (5.79–6.47)
Sex			
Female	–	Reference	Reference
Male	–	1.75 (1.68–1.82)	1.79 (1.72–1.86)
Race			
White	–	Reference	Reference
Black	–	1.10 (1.05–1.16)	1.01 (0.96–1.07)
Hispanic	–	0.79 (0.74–0.86)	0.81 (0.75–0.88)
Other	–	0.66 (0.59–0.73)	0.70 (0.63–0.78)
Median household income			
Above 50th percentile	–	Reference	Reference
Below 50th percentile	–	1.61 (1.54–1.68)	1.47 (1.40–1.53)
Primary discharge diagnosis			
Bipolar disorders	–	–	Reference
Depressive disorders	–	–	1.19 (1.15–1.24)
Psychiatric comorbidities			
None			Reference
Anxiety disorders	–	–	1.10 (1.06–1.14)
Trauma-related disorders	–	–	1.22 (1.17–1.28)
Neurodevelopmental disorders	–	–	0.79 (0.73–0.85)
Tobacco-related disorders	–	–	1.31 (1.26–1.37)
Alcohol-related disorders	–	–	0.85 (0.81–0.89)
Substance-related disorders	–	–	0.95 (0.91–0.99)
Medical comorbidities			
None			Reference
Hypertension	–	–	2.86 (2.75–2.98)
Diabetes	–	–	1.93 (1.82–2.03)
Obesity	–	–	1.54 (1.46–1.62)

## 4. Discussion

Among inpatients with primary diagnoses of mood disorder, comorbid heart disease had a prevalence of 1.3%, predominantly affecting middle-aged men and individuals of White ethnicity. These individuals also exhibited a higher prevalence of both medical and psychiatric comorbidities. Notably, among inpatients with comorbid heart disease, vitamin D deficiency was more frequently detected, resulting in a 26% elevated risk. Nevertheless, when factoring in concurrent medical and psychiatric comorbidities, the associated risk did not achieve statistical significance.

Our study revealed that comorbid heart disease was present in 1.3% of inpatients diagnosed with mood disorders. Extensive literature explores the intricate relationship between mood disorders and cardiovascular health, pointing toward a bidirectional connection linking depression and heart disease (18). Recently, the American Health Association has classified major depressive disorder and bipolar disorder as moderate (“tier-II”) risk conditions for cardiovascular disease (19, 20). Mood disorders have been known to autonomously contribute to the risk of cardiovascular disease, distinct from the impact of conventional risk factors. (21, 22). An estimated 7 million Americans living with CVDs are concurrently experiencing either clinically diagnosed depression or depression symptoms that significantly impair their quality of life. Additionally, this public health burden is compounded by the addition of half a million new cases annually (23). The 12-month prevalence of depression in the general population was 117.0%, while it was 20% among individuals with coronary heart disease (23).

The American Heart Association (AHA) estimates that cardiovascular disease (CVD) affects approximately 40% of individuals aged 40–59 years, 75% of those aged 60–79 years, and 86% of those aged 80 years and above in the US (24). In contrast, major depressive disorder typically begins around the age of 32 years with recurrent episodes lasting for 20 weeks, while bipolar disorders tend to have earlier onset at approximately 18.2 years for bipolar I and 20.3 years for bipolar II (25). Our findings suggest that individuals with mood disorders may have a lower prevalence of comorbid heart disease and older age groups may face a higher risk of cardiac disease among this population, considering the varying rates of mood disorders and comorbid conditions. Our sample consisted solely of inpatients with mood disorders up to the age of 50 years, which could potentially account for the low prevalence observed in our study.

In our study, we observed a substantial burden of medical comorbidities among inpatients with mood disorders who also had comorbid heart disease. It is important to highlight that individuals with serious mental illness are estimated to have a significantly shorter lifespan, with a mortality rate that is 25 years earlier than the general population (26). Approximately 60% of these premature deaths among individuals with serious mental illness are attributed to general medical conditions (26). A study conducted in the United Kingdom, which assessed medical illnesses in bipolar disorder, reported that hypertension was present in 15% of the sample comprising 1720 patients (27). Consistent with this finding, we identified hypertension as the most prevalent medical comorbidity among patients with comorbid heart disease. Moreover, even after adjusting for confounding factors, hypertension emerged as the most influential risk factor in inpatients with comorbid heart disease.

In our sample, the prevalence of vitamin D deficiency in individuals with mood disorders was found to be 2.3%. Notably, a significant difference was observed among those with comorbid heart disease, with a prevalence of 2.9%. Both the unadjusted model and the adjusted model controlled for only demographics demonstrated that vitamin D deficiency increased the risk by 26%. However, in the final regression model when adjusted for potential confounders including comorbidities, the risk of association was not statistically significant. These findings are consistent with a study that also reported no correlation between vitamin D levels and bipolar disorder although a potential connection was hypothesized (28). It has been shown that vitamin D possesses immunomodulatory activity, which can have a positive impact on mood balance (28, 29). Moreover, depression has been linked to cardiovascular disease, and vitamin D deficiency has been hypothesized as a contributing factor to both cardiovascular events and depression (28). While there is some evidence supporting the potential benefits of vitamin D supplementation in managing depression (16), the mixed and conflicting findings emphasize the need for further research and well-designed clinical trials to provide a clearer understanding of its efficacy and the factors that influence its effects on mood disorder (16, 30). A recent study from Italy found that a significant proportion (approximately 80%) of patients with bipolar disorders (BDs) who were hospitalized for a mood episode (manic, hypomanic, depressed, or mixed) exhibited lower than normal vitamin D levels. Their mean vitamin D levels were notably below the recommended normative values, with the majority falling into the categories of insufficient, critical, or severely critical levels. This finding underscores a potential association between BDs and vitamin D deficiency, adding valuable information to the existing literature, which has been limited and inconsistent regarding this relationship.

We did find a high burden of other psychiatric illnesses in our sample. As per a previous study, individuals with a lifetime history of major depressive disorder (MDD) have a high likelihood of experiencing another psychiatric illness during their lives, with nearly 75% being affected. Among these conditions, anxiety disorders are the most prevalent, with approximately 60% of individuals with depression meeting the criteria for an anxiety disorder (31). Additionally, substance-use disorders are observed in up to 24% of individuals with MDD (31). Approximately 75% of patients with bipolar disorder are diagnosed with an anxiety disorder at some point in their lives. Substance-use disorders are present in approximately 42.3% of individuals with bipolar disorder, while impulse control disorders are diagnosed in 62.8% of cases (32). Additionally, tobacco-related disorders were found to have a higher risk of association with mood disorders and comorbid heart disease. Persistent tobacco use is strongly associated with a heightened prevalence of chronic health conditions related to tobacco (33). Individuals who smoke and have either a chronic health condition or a mental health condition are less likely to quit smoking compared to those without any co-occurring conditions (33).

However, the cross-sectional design used in this study limits the ability to establish causality between variables. Longitudinal or experimental designs would provide stronger evidence for causal relationships. Moreover, data from the nationwide inpatient sample (NIS) of non-federal community hospitals may introduce selection bias and limit generalizability to individuals not seeking hospital care or those with different healthcare access. Additionally, the use of administrative data from the NIS may be prone to coding errors or misclassifications, potentially affecting the accuracy and reliability of the findings. While the study adjusted for some demographic variables, unmeasured confounders, such as lifestyle factors or specific treatments, were not accounted for, potentially influencing the observed associations. The study also focused on a selected set of comorbidities, omitting a comprehensive evaluation of all potential conditions that may impact the relationship between mood disorders and comorbid heart disease. Vitamin D deficiency in this study was identified by ICD-10 codes which may lead to under-diagnosis/under-representation due to no additional data on serum vitamin D levels. Looking at the strengths of our study, the study included a substantial number of inpatients with mood disorders, enhancing the statistical power and generalizability of the findings. The use of the nationwide inpatient sample provided data from non-federal community hospitals across multiple states, increasing the diversity and representativeness of the sample. The study employed logistic regression models, adjusting for demographic variables, to explore the associations between comorbid heart disease and various factors, allowing for a more comprehensive analysis.

## 5. Conclusion

In summary, our study emphasizes the prevalence of comorbid heart disease in inpatients with mood disorders and emphasizes the importance of addressing these concurrent conditions. Notably, middle-aged men with depressive disorders and individuals from low-income households face a heightened risk of developing comorbid heart disease. Additionally, trauma-related and tobacco-related disorders contribute to a 20–30% increased risk of comorbid heart disease in inpatients with mood disorders.

Patients presenting both heart disease and mood disorders often exhibit an elevated prevalence of medical and psychiatric comorbidities. Consequently, clinicians should consider routine screenings for comorbid heart disease, especially in individuals with mood disorders and established cardiovascular risk factors such as hypertension, diabetes, obesity, and tobacco use. Addressing and managing these comorbidities is crucial, including addressing the high incidence of trauma, stress, and substance use in these populations.

Vitamin D deficiency was associated with a 26% increased risk of comorbid heart disease in mood disorders, although this association lost significance after adjusting for demographics and cardiovascular risk factors. Nevertheless, we recommend screening for vitamin deficiencies, as early intervention may be beneficial in preventing severe cardiovascular outcomes and worsening mental health issues. The relationship between mood disorders, cardiovascular risk, and vitamin D deficiency warrants further investigation.

Our study highlights the necessity of screening mood disorder patients for medical comorbidities not only in primary care settings but also in mental health clinics. These findings underscore the importance of an integrated approach, involving collaboration between psychiatrists and the medical team, to provide comprehensive care for patients with both mood disorders and cardiovascular conditions.

## Data availability statement

The raw data supporting the conclusions of this article will be made available by the authors, without undue reservation.

## Ethics statement

Ethical approval was not required for the studies involving humans because NIS is a publicly available de-identified dataset from the agency for Healthcare Research and Quality (AHRQ), and institutional review board permission was not required as per the US Department of Health and Human Services. The studies were conducted in accordance with the local legislation and institutional requirements. Written informed consent for participation was not required from the participants or the participants’ legal guardians/next of kin in accordance with the national legislation and institutional requirements because NIS is a publicly available de-identified dataset from the agency for Healthcare Research and Quality (AHRQ), and institutional review board permission was not required as per the US Department of Health and Human Services.

## Author contributions

MH: Writing – original draft. SJ: Conceptualization, Formal analysis, Writing – review & editing, Project administration, Writing – original draft. SP: Writing – original draft. AS: Writing – original draft. AP: Writing – original draft. SV: Writing – original draft. AA: Writing – review & editing. MP: Supervision, Writing – review & editing. AB: Supervision, Writing – review & editing. SR: Supervision, Writing – review & editing. RM: Supervision, Writing – review & editing. RP: Conceptualization, Data curation, Formal analysis, Investigation, Methodology, Resources, Software, Supervision, Validation, Writing – review & editing.
